# Effects of abscisic acid and brassinolide on photosynthetic characteristics of *Leymus chinensis* from Songnen Plain grassland in Northeast China

**DOI:** 10.1186/1999-3110-54-42

**Published:** 2013-10-02

**Authors:** Yong-Jun Hu, Lian-Xuan Shi, Wei Sun, Ji-Xun Guo

**Affiliations:** 1 Key Laboratory for Vegetation Ecology, Ministry of Education, Institute of Grassland Science, Northeast Normal University, Changchun, 130024 China; 2 School of Life Sciences, Changchun Normal University, Changchun 130032 China

**Keywords:** Abscisic acid, Brassinolide, *Leymus chinensis*, Photosynthetic characteristics, Songnen plain grassland

## Abstract

**Background:**

It has been well demonstrated that plant growth regulators have important functions in multiple physiological processes. ABA and BR play crucial roles in response of crops to stresses. Photosynthetic capacity of *Leymus. chinensis* treated by various concentrations of ABA and BR in combination was determined. Further more, the mechanisms of ABA and BR treatments and potential for recovery of saline-alkali grasslands were discussed.

**Results:**

Abscisic acid (ABA) and brassinolide (BR) affected leaf gas exchange, growth and biomass of *L. chinensis*. The application of ABA and BR mixtures, especially that of 0.01 mM ABA and 2 × 10^-4^ mM BR, increased the net photosynthetic rate, stomatal conductance, water use efficiency, the maximum net photosynthetic rate, light-saturated rate, leaf respiration rate, the maximum RUBP carboxylation rate, the maximum electron transport rate, the maximum triose-phosphate utilization, carboxylation efficiency and the quantum efficiency of PSII and subsequently enhanced density, height and biomass in *L. chinensis*. We also observed reduction in the light compensation and saturation points following the application of ABA and BR treatments.

**Conclusions:**

We concluded that proper use of plant growth regulators can enhance the plant growth and productivity on the Songnen grassland, which is particularly important for the improvement of saline – alkaline grassland and the yield of grazing lands.

**Electronic supplementary material:**

The online version of this article (doi:10.1186/1999-3110-54-42) contains supplementary material, which is available to authorized users.

## Background

It has been well demonstrated that plant growth regulators are involved in multiple physiological processes Krouk et al. (2011). Plant growth regulators are increasingly used for the improvement of plant growth and stress resistance. Recent publications reported the effects of several resistance-related hormones, including salicylic acid, jasmonates, polyamines, and 5-aminolevulinic acid (ALA), etc. on plant physiological activities. As the most studied stress-responsive hormone, abscisic acid (ABA) play crucial roles in response of plants to abiotic stresses such as drought, salinity and frost Wu (2010). For the water-stressed plants, ABA can decrease water loss via transpiration, improve the antioxidant enzymes system, and induce the expression of stress-related genes. Moreover, exogenous application of ABA significantly influences leaf photosynthesis and photosynthate accumulation through regulating stomata openness and/or activities of photosynthetic enzymes. ABA treatments show complex effects on leaf photosynthesis. Šafránková et al. (2007) found that ABA treatment significantly decreased the net photosynthetic rate (*P*_N_) and transpiration rate (*E*) of the water-stressed barley. ABA associated decrease in *P*_N_ was also be found in *Stylosanthes guianensis* Zhou et al. (2006) and *Pennisetum typhoides* Sankhla and Huber (1974). However, several studies showed positive effects of ABA treatment on leaf photosynthesis McLaren and Smith (1977; Jia and Lu 2003; Li et al. 2006). The compromise results were also be found by Mawson et al. (1981), and Franks and Farquhar (2001). The above mentioned inconsistent results about the effects of ABA on leaf gas exchange may be caused by multiple factors, including differences in stress factors, ABA dosage and treatment time McLaren and Smith (1977).

In addition to the inconsistent effects of plant hormone on plant growth performance, the mixture of different plant hormones can produce additive action or counteraction on plant metabolism Peleg and Blumwald (2011). For example, Sankhla and Huber (1974) found that the mixture of abscisic and gibberellic acids tended to antagonize each other in incorporation of ^14^CO_2_ into photosynthetic products. Brassinolide (BR), another common plant hormone with high biological activity, is found recently in vegetables Khripach et al. (2000). BR treatments have significant effects on plant growth and stress resistance Bajguz and Hazat (2009). BR treatments lead to the increase of net photosynthetic rates Vardhini and Ramr (1998 Hou and Li 2001) or delay the reduction of photosynthetic rate Liu et al. (2008). However, the information on the influence of ABA and BR in combination on leaf photosynthesis is less available.

To date, most studies about ABA and BR are mainly focussed on crops with very few studies on perennial grasses. *Leymus chinensis,* a perennial grass, is the dominate species in the salinized Songnen grassland in Northeastern China Li and Zheng (1997). The Songnen grassland covers approximately 20–25% of the total area in Songnen plain and is mainly utilized for hay production and livestock grazing Li and Zheng (1997). The present study was conducted over three years within self-sown *L. chinensis* populations. Photosynthetic capacity of *L. chinensis* treated by various concentrations of ABA and BR in combination was determined. Furthermore, the mechanisms of ABA and BR treatments and potential for recovery of saline-alkali grasslands were discussed.

## Methods

### Study site and experimental design

This research was conducted at The Grassland Ecosystem Experimental Station of Northeast Normal University, Chang Ling Horse Breeding Farm in Jilin Province (44°30′ to 44°45′N, 123°31′ to 123°56′E), Northeast China. The study area has a typical mesothermal monsoon climate, with an altitude of 37.8 to 144.8 m. The region is cold and dry in spring with frequent wind, warm and wet in summer with frequent drought, early frosts in autumn, and long, cold winters with little snowfall. The mean annual temperature is 5.0°C with a frost–free period of 136 to 146 d. The mean annual precipitation is about 450 mm mainly occured from June to August and accounts for over 60% of the annual precipitation. The annual evaporation is 2 to 3 times higher than precipitation. Salinized meadow soil is the main soil type in the Songnen grassland.

Seven plots were selected for sampling. Each plot area was 10 × 10 m with a 2 m isolation belt between plots. The *L. chinensis* community in the selected area had been established for two years by artificial seeding. The plants of *L. chinensis* were uniform in size with almost no weeds. Seven treatments were applied (Table 1).

**Table 1:** **Experimental treatments**

Treatment	Concentration(mM)
Control	——
ABA	0.01
High BR	2 × 10^-4^
ABA + low BR	0.01, 0.02 × 10^-4^
ABA + medium BR	0.01, 0.2 × 10^-4^
ABA + high BR	0.01, 2 × 10^-4^
High ABA + high BR	0.1, 2 × 10^-4^

ABA*:* abscisic acid; BR: brassinolide.

ABA and BR were sprayed during the middle ten days of May in 2005 and 2006, respectively. Fully expanded leaves of plants from each plot were used to measure photosynthetic characteristics. The density, height, and biomass of the *L. chinensis* community were determined using standard sampling methods Shi and Guo (2006), and each measurement was repeated 5 times.

*P*_N_, *g*_s_, *C*_i_/*C*_a_, and *E* were determined using a portable open flow gas exchange system LI–6400XT (*LI-COR*, USA) at 2 h intervals from 8:00 h to 16:00 h. WUE was calculated as *P*_N_/*E*. The photosynthetically active radiation (PAR) was 1000 ± 12 μmol m^-2^ s^-1^, CO_2_ concentration was 350 ± 2 ppm, and leaf temperature was 26.0 ± 0.8°C. Gas exchange was measured on fully expanded leaves from the same adult plants for five plants per plot. Measurements were repeated three times for each selected plant. Moreover, measurements were done within three consecutive days in mid-July.

### The responses of photosynthesis to light (*A*/*Q*)

For the measurement of *A*/*Q*, the photosynthetic photon flux density levels used for the construction of light response curves were: 2000, 1800, 1600, 1400, 1200, 1000, 800, 600, 400, 200, 100, 50 and 0 μmol m^-2^ s^-1^ generated by a LI-6400/02B red/blue light source Wu *et al*. (2007). The CO_2_ concentration was kept at 380 μmol mol^-1^ Wang and Zhou (2004). Each measurement was repeated 10 times between 9:00 h to 12:00 h in mid-July. All *A*/*Q* parameters were determined by fitting data to the quadratic equation described by Prioul and Chartier (1997). Q_E_, LCP, LSE, *A*_max_ were modeled using the linearization of *A*/*Q* curves (Long and Bernacchi 2003).

### The responses of photosynthesis to CO_2_(*A*/*C*_i_)

The same process used in *A*/*Q* measurements was used for *A*/*C*_i_ curves. A range of CO_2_ concentrations (*C*_**i**_), i.e. 1800, 1600, 1400, 1200, 1000, 800, 600, 400, 200, 100 and 50 μmol mol^-1^ was generated using a 12 g CO_2_ cylinder, starting from 1800 and ending at 50 μmol mol^-1^. The *A*/*C*_i_ curves were measured under four light gradients: 1200, 1000, 800 and 400 μmol m^-2^ s^-1^Dordas and Sioulas (2007). Leaf temperature was kept at 32 ± 0.8°C and each gradient of light or CO_2_ concentration treatment was repeated five times between 9:00 h and 10:00 h. *A*_sat_, CCP, *Resp*, *V*_cmax_, *J*_max_, *V*_TPU_ and *CE* were calculated according to Prioul and Chartier (1997) and Olsson and Leverenz (1994). Leaves were held in the chamber until values of photosynthesis reached a steady state. At CO_2_ concentration, the analysis of photosynthetic response to CO_2_ Field et al. (1989, Chen *et al*. 2006) was accompanied by a match. A model curve described by the rectangular hyperbola Olsson and Leverenz (1994) was fitted.

### Chlorophyll fluorescence

Chlorophyll fluorescence measurements included initial fluorescence (F_o_), maximum fluorescence (F_m_) and variable fluorescence (F_v_). F_o_ refers to fluorescence when PSII reaction center opens entirely. The decrease of F_o_ indicates the increase of antenna hot dissipation; and the increase of F_o_ indicates the uneasy reversing damage of PSII reaction center. F_m_ refers to fluorescence when PSII reaction center closes entirely. A decrease in F_m_ indicates inhibition of photosynthesis. F_v_, the difference between F_m_ and F_o_, reflects a reduction in Q_A_. The first fully expanded, healthy leaves were measured using Li–6400XT (LI-COR, USA) for dark-acclimated and light- acclimated measurements. F_o_ was measured after 20 min of dark acclimation during a low intensity pulsed. F_m_ was recorded in a 0.8 s pulse of saturating light (6500 μmol m^-2^ s^-1^).

### Statistical analyses

All photosynthetic parameters were analyzed using SPSS (v. 11.0 for Windows, USA) and *SigmaPlot* 10. Photosynthesis Assistant was used to analyze parameters related to responses to light and CO_2_. The level of statistical significance was *P* ≤ 0.05.

## Results

### Quantitative changes in plant growth parameters and plant density

**Figure 1:**
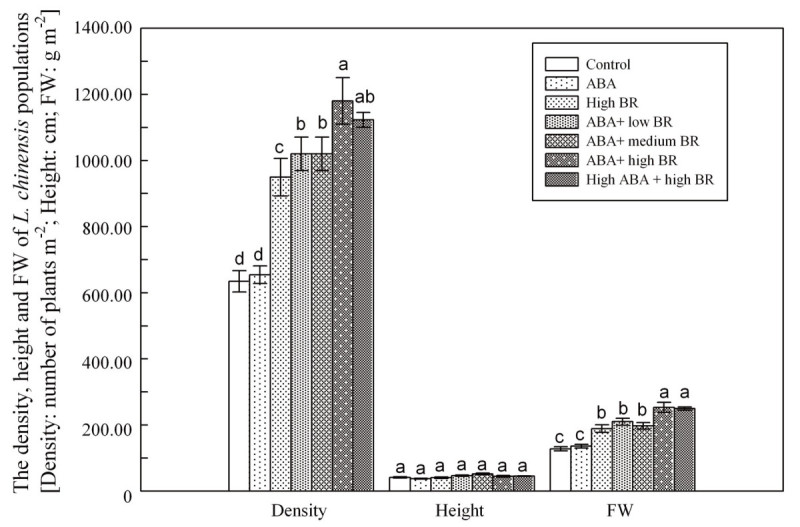
**Changes in the density, height and the FWof** ***L. chinensis*** **populations under ABA and BR treatments (Density: number of plants m** ^**-2**^ **; Height: cm; FW: fresh weight g m** ^**-2**^ **).**

**Figure 2:**
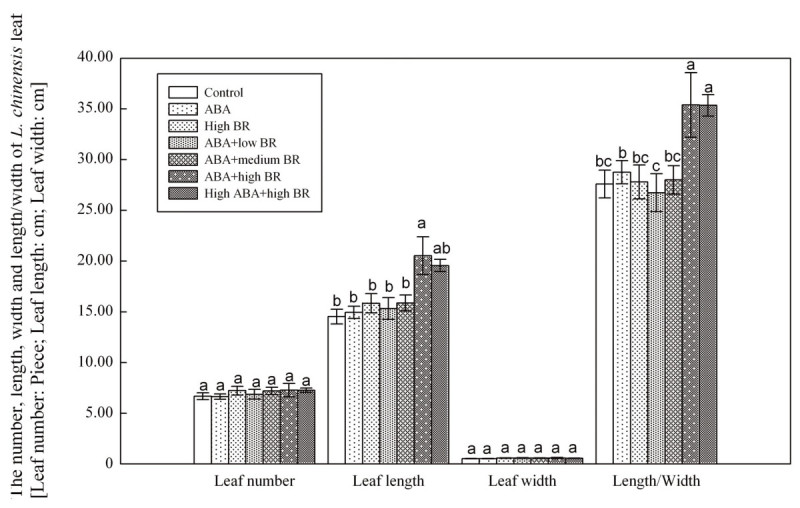
**Changes in the number, length, width and length/width of** ***L. chinensis*** **leaves under ABA and BR treatments (Leaf number: piece; Length: cm; Width: cm).**

Plant density, height, and fresh mass of *L. chinensis* were significantly affected by ABA and BR treatments (Figure 1). Plant density increased, compared to control, 49.6%, 60.7%, 60.7%, 85.9% and 76.9% at 2 × 10^-4^ mM BR, 0.01 mM ABA and 0.02 × 10^-4^ mM BR, 0.01 mM ABA and 0.2 × 10^-4^ mM BR, 0.01 mM ABA and 2 × 10^-4^ mM BR, 0.1 mM ABA and 2 × 10^-4^ mM BR treatments, respectively. Plant height increased 12.7%, 25.7%, 7.7%, and 9.2% , respectively at 0.01 mM ABA and 0.02 × 10^-4^ mM BR, 0.01 mM ABA and 0.2 × 10^-4^ mM BR, 0.01 mM ABA and 2 × 10^-4^ mM BR, 0.1 mM ABA and 2 × 10^-4^ mM BR treatments. However, plant height decreased 9.4% and 1.8% at ABA alone and BR alone treatments compared to the control. Plant biomass of the six respective treatments significantly increased 6.5%, 48.2%, 64.7%, 54.6%, 98.6%, 95.8% compared to the control. Various ABA and BR treatments showed significant differences (*P* ≤ 0.05) among treatments for *L. chinensis* leaf characters (Figure 2). For the leaves of single plant, results were in accordance with those for community plant biomass. The length and length/width ratio were significantly different (*P* ≤ 0.05) for 0.01 mM ABA and 2 × 10^-4^ mM BR, 0.1 mM ABA and 2 × 10^-4^ mM BR treatments, which increased length by 41.3% and 34.7%, respectively, and width by 28.3% and 28.2%,respectively, compared to control.

### Diurnal patterns of leaf gas exchange

**Figure 3:**
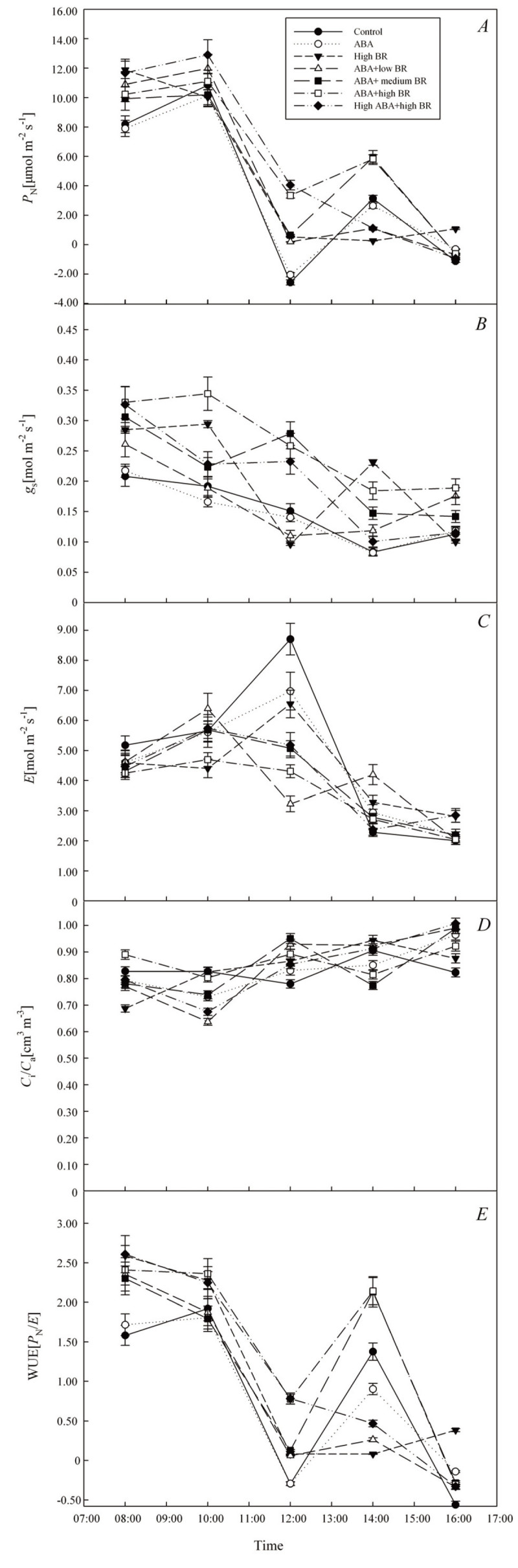
**Daily changes in photosynthetic parameter of** ***L. chinensis*** **under ABA and BR treatments.** **(** ***A*** **)** net photosynthetic rate, *P*_N_, **(** ***B*** **)** stomatal conductance, *g*_s_, **(** ***C*** **)** transpiration rate, *E*, **(** ***D*** **)** ratio of stomatal and sub-stomatal CO_2_ concentrations, *C*_i_/*C*_a_, and **(** ***E*** **)** water use efficiency, WUE.

Diurnal patterns in *P*_N_ were similar among treatments and exhibited bimodal curves, reflecting a significant drop at noon (Figure 3*A, P* ≤ 0.05). The daily average values of *P*_N_ showed a upward trend. All treatments were significant higher than the control except the treatment of ABA alone. Notably, the 0.01 mM ABA and 2 × 10^-4^ mM BR treatment had the highest *P*_N_ values among the treatments. There were no apparent differences in diurnal patterns in *g*_s_ among the treatments. However, the average value of *g*_s_ in ABA and BR treatments were significantly (*P* ≤ 0.05) higher than the control. Especially, 0.01 mM ABA and 2 × 10^-4^ mM BR treatments was significantly (*P* ≤ 0.05) higher than the control and other treatments (each increased on average by 74.0%, 45.0%, 29.2%, 52.2%, 18.6%, 29.6%). (Figure 3*B*). Diurnal patterns in *C*_i_**/** *C*_a_ did not differ significantly among treatments, presenting a general stable trend (Figure 3*C*). The daily average value of *C*_i_/*C*_a_ also did not differ significantly among treatments. The diurnal patterns of *E* varied greatly among treatments, and at 12:00 h, the hormone treatment significantly (*P* ≤ 0.05) decreased the transpiration rate of *L. chinensis* leaves (Figure 3*D*). The daily average value of *E* showed a declining trend from ABA alone treatment to ABA and high BR treatment. High ABA and high BR treatment increased *E* but still lower than the control. Each of the treatments was on average lower by 2.1%, 9.6%, 14.0%, 15.9%, 24.4%, and 13.5% relative to the control. The pattern of daily WUE was similar to those of *P*_N_, showing a bimodal curve. At 14:00 h, the differences among treatments in WUE were the largest, and WUE of 0.01 mM ABA and 0.2 × 10^-4^ mM BR, and 0.01 mM ABA and 2 × 10^-4^ mM BR treatments were significantly (*P* ≤ 0.05) higher than the other treatments (Figure 3*E*). The daily mean WUE of *L. chinensis* increased due to treatments, except ABA alone. Treatment of 0.01 mM ABA and 2 × 10^-4^ mM BR showed the highest WUE, with 80.3%, 97.3%, 34.1%, 72.1%, 19.5%, and 25.5% increases over other treatments.

### Photosynthetic response to light (*A*/*Q*)

Significant differences in photosynthetic parameters were observed among ABA and BR treatments with different concentrations (Table 2). The Q_E_ values of *L. chinensis* were increased in treatments compared to the control, 0.01 mM ABA and 2 × 10^-4^ mM BR, 0.1 mM ABA and 2 × 10^-4^ mM BR treatments were significantly higher than others. The *A*_max_ of 0.01 mM ABA was lower than the control; the other treatments were higher than the control plots, in particular, 0.01 mM ABA and 2 × 10^-4^ mM BR treatment increased *A*_max_ by 63.2%. However, LCP and LSE trended to decrease, especially for 0.01 mM ABA and 2 × 10^-4^ mM BR, 0.1 mM ABA and 2 × 10^-4^ mM BR treatments. The results showed that hormone treatments influenced photosynthetic responses to light.

**Table 2:** **Photosynthetic parameters of** ***L. chinensis*** **in response to light under ABA and BR treatments**

Treatment	***Q***_***E***_	***A***_***max***_[μmol m^–2^ s^-1^]	***LCP***	***LSE***
Control	0.02 ± 0.00d^1^	10.33 ± 0.93c	78.66 ±1.85a	502.80 ±10.77a
ABA	0.04 ± 0.00c	9.96 ± 0.85c	52.96 ± 1.94b	283.10 ± 9.56b
High BR	0.07 ± 0.00b	13.26 ± 0.67bc	52.28 ± 1.25b	255.20 ± 8.74b
ABA + low BR	0.06 ± 0.00b	13.88 ± 0.53bc	54.21 ± 1.68b	295.80 ± 10.65b
ABA + medium BR	0.07 ± 0.00b	13.64 ± 0.78bc	45.08 ± 1.34c	548.40 ± 11.28a
ABA + high BR	0.11 ± 0.00a	16.86 ± 0.91a	25.07 ± 1.25d	175.70 ± 10.34c
High ABA + high BR	0.11 ± 0.00a	14.14 ± 0.94b	24.58 ± 0.99d	157.00 ± 10.26c

(ABA: abscisic acid; BR: brassinolide; *Q*_*E*_: high energy state quenching; *A*_max_:the maximum net photosynthesis; LCP: light compensation point; LSE: light saturation estimate. ^1^ : Different letters with a column indicate significate difference at *P* ≤ 0.05 ).

### The responses of photosynthesis to CO_2_(*A*/*C*_i_)

CO_2_ compensation point (CCP) were not affected by treatments, but there were significant differences in *A*_sat_, *Resp*, *V*_cmax_, *J*_max_, *V*_TPU_, *CE* among treatments (Table 3). *A*_sat_ and *Resp* showed no obvious change occurred between the ABA and the BR treatments versus the control, but the values under ABA and BR combined treatments were higher than that of the control, especially the 0.01 mM ABA and 2 × 10^-4^ mM BR treatment, increased *A*_sat_ and *Resp* by 58.9% and 37.7%, respectively. *V*_cmax_ and *CE* showed similar patterns to *A*_*sat*_: with limited influence by ABA and BR treatments alone. However, the ABA and BR combined treatments affected *V*_cmax_ and *CE* significantly (*P* ≤ 0.05), especially for 0.01 mM ABA and 2 × 10^-4^ mM BR, and 0.1 mM ABA and 2 × 10^-4^ mM BR treatments. *J*_max_ was higher in all treatments than the control, especially 0.01 mM ABA and 2 × 10^-4^ mM BR. *V*_TPU_ was higher in all treatments than the control except 0.01 mM ABA and 0.2 × 10^-4^ mM BR; 0.1 mM ABA and 2 × 10^-4^ mM BR gave the largest increase among treaments.

**Table 3:** **Photosynthetic parameters of** ***L. chinensis*** **in responses to CO**_**2**_ **(** ***C***_**i**_ **) under ABA and BR treatments**

Treatment	***A***_sat_	CCP	***Resp***	***V***_cmax_	***J***_max_	***V***_TPU_	***CE***
	[μmol m^–2^ s^-1^]						
Control	31.16 ± 3.61 cd^1^	30.00a	18.30 ± 1.15c	33.50 ± 2.82de	140.00 ± 2.67e	22.70 ± 1.67c	0.04 ± 0.00c
ABA	27.57 ± 4.25d	30.00a	18.40 ± 1.29c	33.50 ± 2.30de	162.00 ± 2.81d	28.20 ± 1.29b	0.04 ± 0.00c
High BR	31.27 ± 2.56 cd	30.00a	17.20 ± 1.05 cd	34.10 ± 2.64d	191.00 ± 3.11c	32.70 ± 1.87a	0.04 ± 0.00c
ABA + low BR	32.06 ± 3.17c	30.00a	23.10 ± 2.00ab	37.90 ± 2.71c	199.00 ± 3.09c	27.00 ± 1.48b	0.07 ± 0.00b
ABA + medium BR	33.19 ± 3.89c	30.00a	24.60 ± 1.65a	45.20 ± 2.35b	237.00 ± 3.47b	20.40 ± 1.95d	0.13 ± 0.00ab
ABA + high BR	49.51 ± 4.06a	30.00a	25.20 ± 1.45a	51.90 ± 2.88a	262.00 ± 3.55a	25.90 ± 1.55bc	0.15 ± 0.00a
High ABA + high BR	41.93 ± 3.73b	30.00a	20.80 ± 1.82b	51.10 ± 2.63a	236.00 ± 3.17b	32.80 ± 1.73a	0.14 ± 0.00ab

ABA: abscisic acid; BR: brassinolide; *A*_sat_: light-saturated rate of net photosynthesis; CCP: CO_2_ compensation point; *Resp:* respiration; *V*_cmax_: maximum RUBP carboxylation rates; *J*_max_: the maximum electron transport; *V*_TPU_: maximum triose-phosphate utilization; *CE*: carboxylation efficiency calculated from the data of photosynthetic response to CO_2_. ^1^ : Different letters with a column indicate significate difference at *P* ≤ 0.05.

### Chlorophyll fluorescence

**Figure 4:**
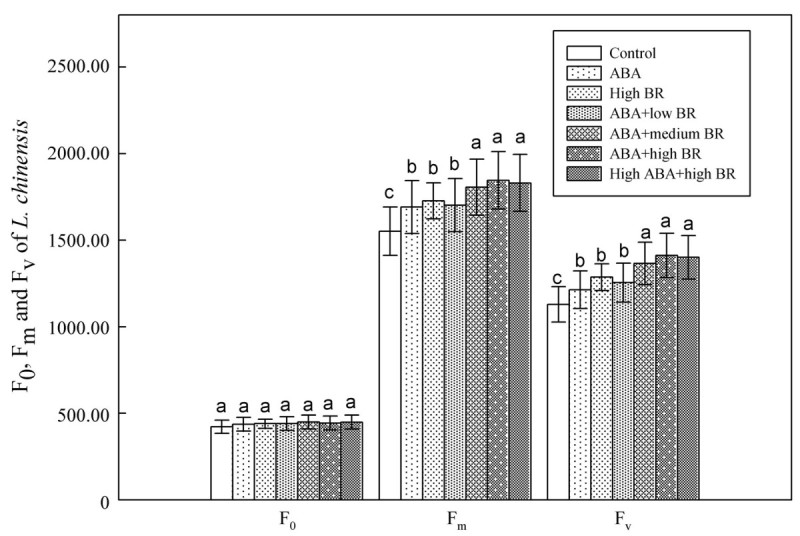
**Changes in F**_**o**_**, F**_**m**_**and F**_**v**_**of** ***L. chinensis*** **under ABA and BR treaments (F**_**o**_**: initial fluorescence; F**_**m**_**: maximum fluorescence; F**_**v**_**: variable fluorescence)**.

**Figure 5:**
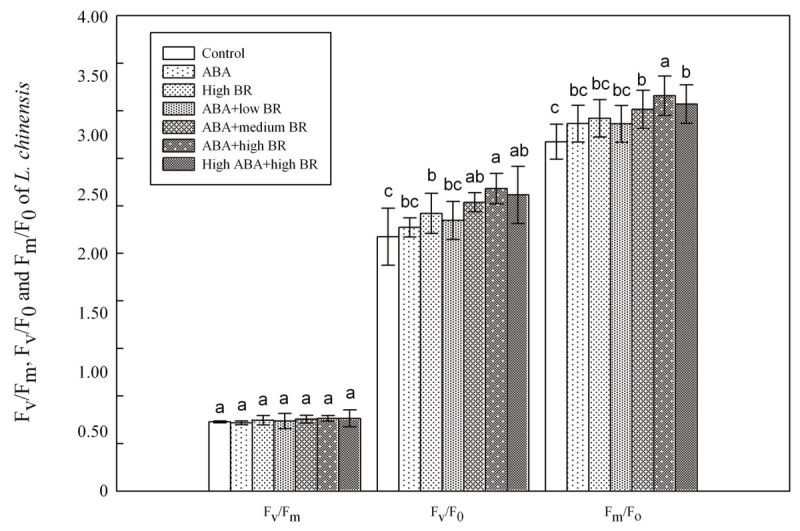
**Changes in F**_**v**_ **/F**_**m**_ **; F**_**v**_ **/F**_**o**_ **and F**_**m**_ **/F**_**o**_ **of** ***L. chinensis*** **under ABA and BR treaments (F**_**v**_ **/F**_**m**_ **: maximum quantum yield of PSII photochemistry; F**_**v**_ **/F**_**o**_ **: potential activityof PSII; F**_**m**_ **/F**_**o**_ **: electronic transfer efficiency of PSII)** **.**

F_o_ was not significantly affected by treatments, but significant (*P* ≤ 0.05) differences in F_m_ and F_v_ were observed (Figure 4). F_m_ and F_v_ tended to increase from ABA alone to high ABA and high BR treatments, which were higher than the control. ABA alone, high BR, and 0.1 mM ABA and 2 × 10^-4^ mM BR were higher than the others in F_m_ and F_v_. ABA caused a significant (*P* ≤ 0.05) decrease in F_v_/F_m_ and BR caused a significant (*P* ≤ 0.05) increase in F_v_/F_m_ compared to the control. When the two hormones were applied together, the ABA effect was counteracted by BR, and F_v_/F_m_ showed higher values than when BR was applied alone. ABA alone, BR alone and high ABA and high BR treatments were significantly (*P* ≤ 0.05) higher (Figure 5) . F_v_/F_o_ patterns strongly resembled that of F_m_/F_o_. F_m_/F_o_ expresses the basal quantum yield of non-photochemical processes. From Figure 5, there were no notable differences between the control and treatments. All these results indicated that hormone treatments change the chlorophyll fluorescence of *L. chinensis* leaves and hence influence the photosynthesis.

## Discussion and conclusions

At present, global environment and crop production research lend special significance to improving plant photosynthesis, plant production and biomass through enhancing plant’s ability to resist saline-alkaline, drought and other environment stresses. Amongst others, plant growth regulators and related compounds have shown beneficial functions on the enhancement of plant growth performance and great potential to help realize those above mentioned goals.

Our experiment results indicated that the 0.01 mM ABA treatment inhibited leaf photosynthetic rate, stomatal conductance, and transpiration rate of mature *L. chinensis* to a certain extent, which are consistent with the results of former studies with inhibition of ABA on leaf gas exchange have been observed in several plant species Vardhini and Ramr (1998 Hou and Li 2001). We also observed that the application of 0.01 mM ABA treatment reduced the plant height and leaf quantity of the studied *L. chinensis* populations. However, it increased plant density, fresh mass, plant length and length width ratio of *L. chinensis* populations. Similar results have been reported by Saab et al. (1990) on the effects of ABA application on the growth of maize seedlings. Saab et al. (1990) found that ABA could promote the growth of maize seedling root, and inhibit stem and leaf growth under the water stress or water shortage condition. Moreover, we also found the special physiological effect of ABA treatment alone on other leaf photosynthetic parameters, such as an increase in the maximum RUBP carboxylation rate, leaf respiration rate, maximum electron transport, and maximum triose-phosphate utilization rate of *L. chinensis* leaves and a reduction in the light compensation point and light saturation estimates. The results of leaf chlorophyll fluorescence measurements suggest that ABA treatment enhanced anti-photoinhibition and the ability to resist harmful environments in *L. chinensis*. Overall, despite slightly reduction in plant height and leaf number per shoot, the gas exchange and chlorophyll fluorescence data indicate that the application of ABA alone enhanced leaf photosynthetic activities and CO_2_ assimilation rate.

BR is another important plant growth regulator, which has profound impacts of leaf photosynthesis and plant performance. The results of previous experiments suggest that BR improve leaf carbon assimilation rate through increasing the content of chlorophyll, which is the light harvesting machine of plant photosynthesis. Moreover, it has also showed that BR application could significantly alleviate the impacts of various abiotic stresses. For instance, BR treatment enhanced photosynthetic performance of cotton seedlings under NaCl stress Ding et al. (1995; Xiao et al. 2007; Chen et al. 2007; Shu et al. 2011). For cucumber seedlings, BR treatment has also been found to promote the occurrence of new roots and the formation of lateral roots Bao et al. (2004). Similar results were obtained in our experiment; BR treatment (2 × 10^-4^ mM) alone increased the photosynthetic carboxylation capacity and CO_2_ assimilation rate. Subsequently, BR treatment enhaned the plant density, height and biomass of the studied *L. chinensis* populations. As a saline alkali grassland rhizomatous plant, the occurrence of new and lateral roots is conducive for the growth as well as rhizome breeding of *L. chinensis*. The observed significant treatment effects of BR on *L. chinensis* may attribute to the stimulation of BR on the formation of new and lateral roots, which will not only directly enhance rhizome breeding and population density, but also indirectly improve plant water and nutrient uptake.

The effects of ABA or BR alone treatment on leaf gas exchange and plant performance have been conducted in various plants. However, the impacts of ABA and BR mixture on plant growth have been rarely tested, especially in perennial grasses. We studied the combined impacts of various ABA and BR mixtures on the leaf carboxylation capacity and growth performance of *L. chinensis*. The experimental results showed that ABA and BR treatments mixed in different proportions are evidently superior to treatments with ABA alone and BR alone on the enhancement of photosynthetic assimilation capacity and growth performance. The ABA and BR mixture treatments expressed not simply add up of the impacts of ABA and BR treatment alone, but showed compensatory effects between ABA and BR. This phenomenon is especially significant for the mixture of 0.01 mM ABA and 2 × 10^-4^ mM BR, which evidently increased *P*_N_, *g*_s_ and WUE, as well as *A*_max_, *A*_sat,_*R* esp, *V*_cmax_, *J*_max_, *V*_Tpu_, *CE* and quantum efficiency of PSII, and reduced the LCP and LSE. As a result of enhancement in photosynthetic capacity and CO_2_ assimilation rate, plant density, height and biomass were significantly increased in *L. chinensis*. Despite unclear in the underlying physiological mechanisms of the impacts of ABA and BR treatments on leaf photosynthetic capacity, the observed obvious effects of ABA and BR mixture on plant performance may attribute, to some extent, the compensatory impacts of ABA and BR treatment. For instance, BR application could improve root system and nutrient uptake, whereas ABA treatment enhanced photosynthetic capacities.

This experiment studied the impacts of ABA alone, BR alone and various mixture of ABA and BR on the performance of *L. chinensis*. Treatments of ABA alone or BR alone enhanced plant photosynthetic capacity and growth performance, however those effects were more significant when ABA and BR were implied in mixture. We proposed that there are compensatory effects between ABA and BR on regulating plant photosynthetic capacity and growth performance. Moreover, the experimental results provides evidence for enhancing the stress resistance of perennial plant populations and also builds a basis for recovering grasses growing under saline and alkaline conditions.

## Authors’ original submitted files for images

Below are the links to the authors’ original submitted files for images. Authors’ original file for figure 1 Authors’ original file for figure 2 Authors’ original file for figure 3 Authors’ original file for figure 4 Authors’ original file for figure 5

## Abbreviations

ABA: Abscisic acid
Amax: The maximum net photosynthetic rate
Asat: Light-saturated rate of net photosynthesis
BR: Brassinolide
Ca: Atmospheric CO_2_ concentration
Ci: Intercellular CO_2_ concentration
CCP: CO_2_ compensation point
CE: Carboxylation efficiency calculated from CO_2_ response curve
E: Transpiration rate
Fo: Initial fluorescence
Fm: Maximum fluorescence
Fv: Variable fluorescence
gs: Stomatal conductance
Jmax: The maximum electron transport rate
LCP: Light compensation point
LSE: Light saturation estimate
PN: Net photosynthetic rate
QA: Primary electron acceptor
QE: High energy state quenching
Resp: Respiration
Vcmax: Maximum RUBP carboxylation rate
VTPU: Maximum rate of triose- phosphate utilization
WUE: Water use efficiency.
