# Supplementary figures and images for: Effects of abscisic acid and brassinolide on photosynthetic characteristics of Leymus chinensis from Songnen Plain grassland in Northeast China
Source: Bot Stud. 2013 Oct 2;54:42. doi: 10.1186/1999-3110-54-42 (PMC5430352; doi:10.1186/1999-3110-54-42)

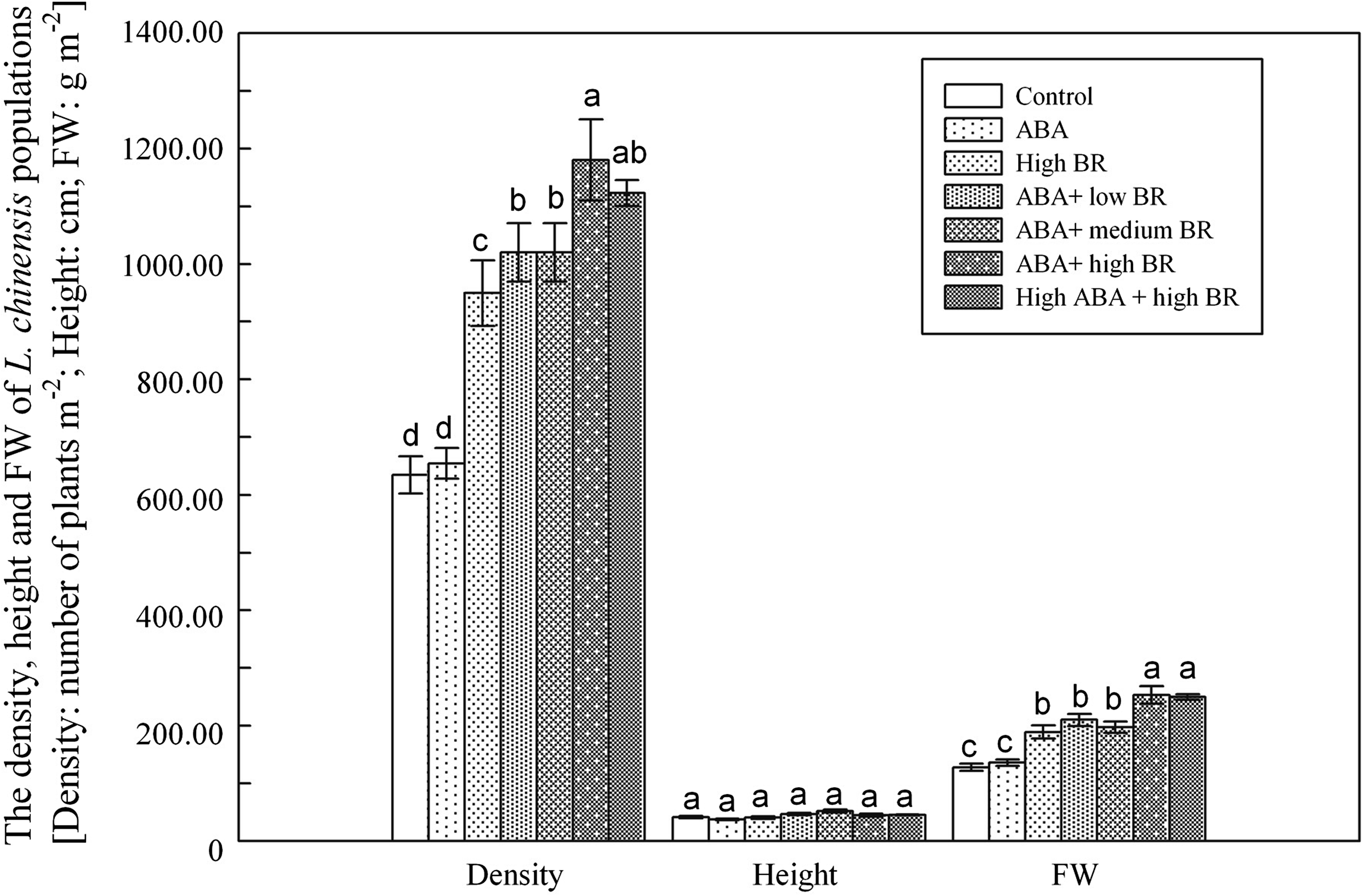

Supplement: Supplementary file 1 — Authors’ original file for figure 1 [file 40529_2011_94_MOESM1_ESM.tiff]

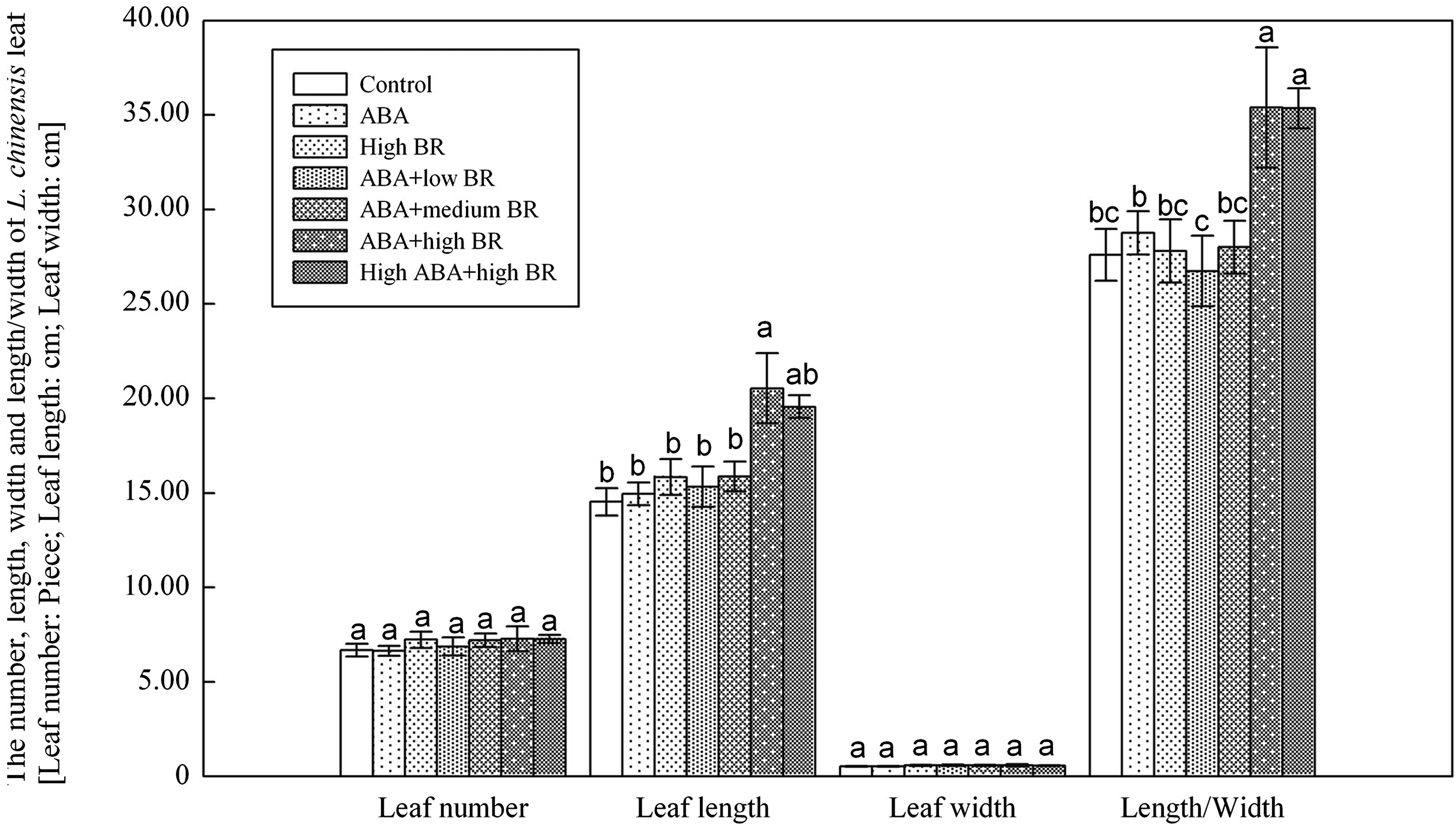

Supplement: Supplementary file 2 — Authors’ original file for figure 2 [file 40529_2011_94_MOESM2_ESM.tiff]

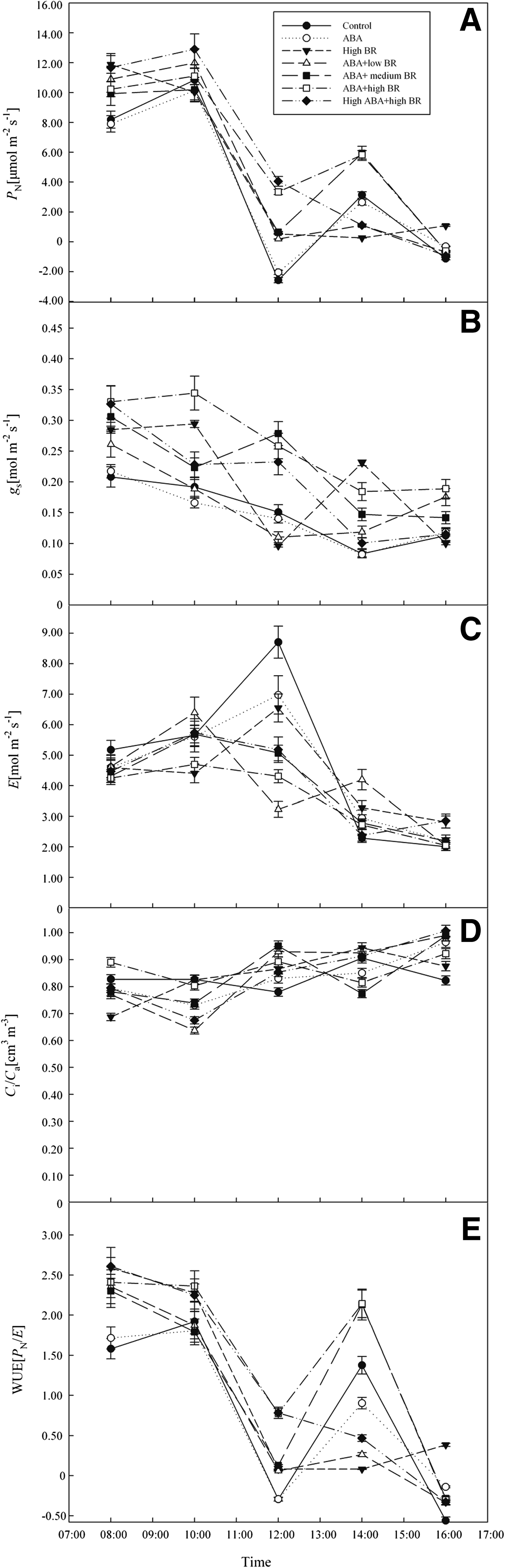

Supplement: Supplementary file 3 — Authors’ original file for figure 3 [file 40529_2011_94_MOESM3_ESM.tiff]

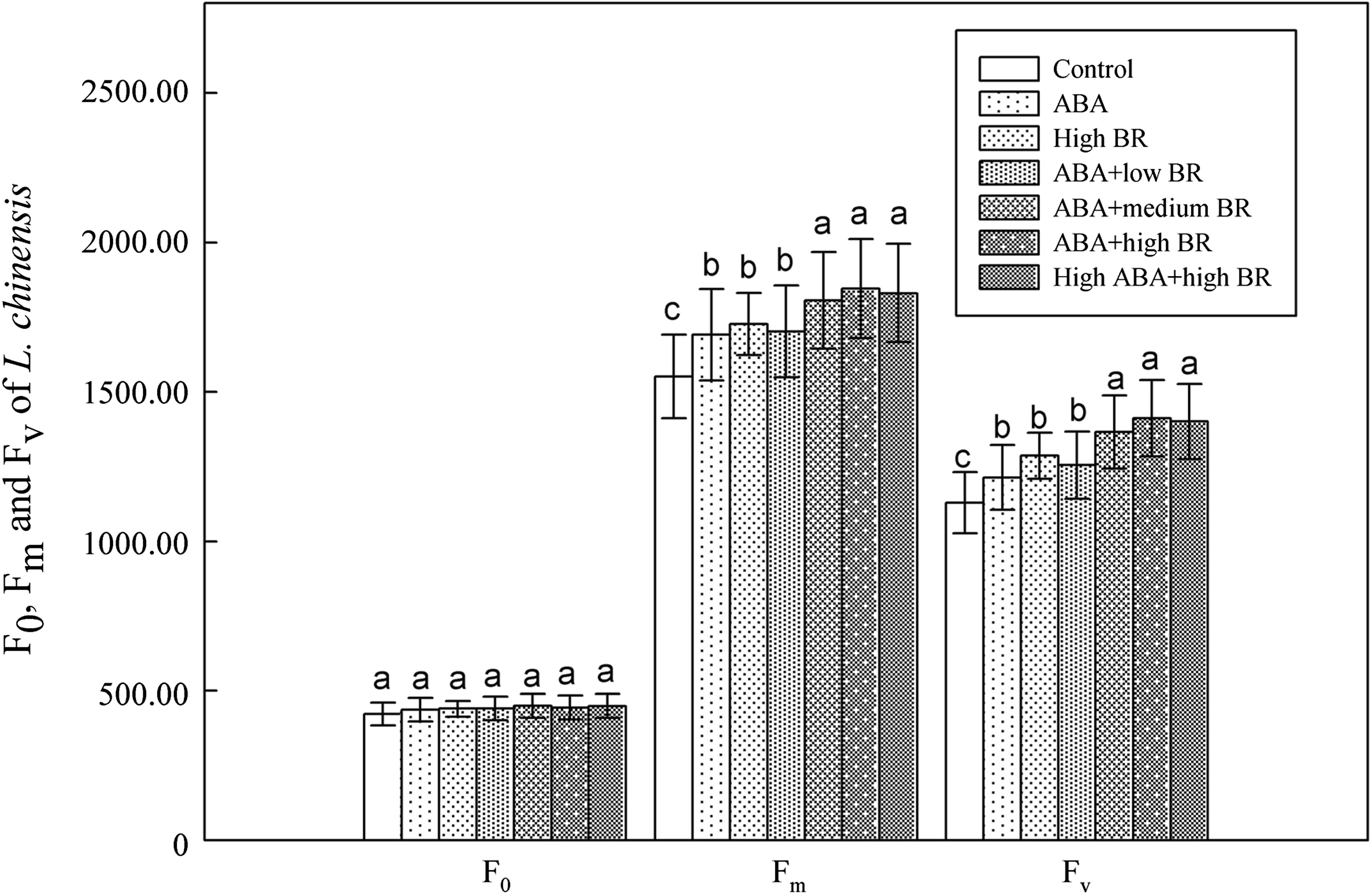

Supplement: Supplementary file 4 — Authors’ original file for figure 4 [file 40529_2011_94_MOESM4_ESM.tiff]

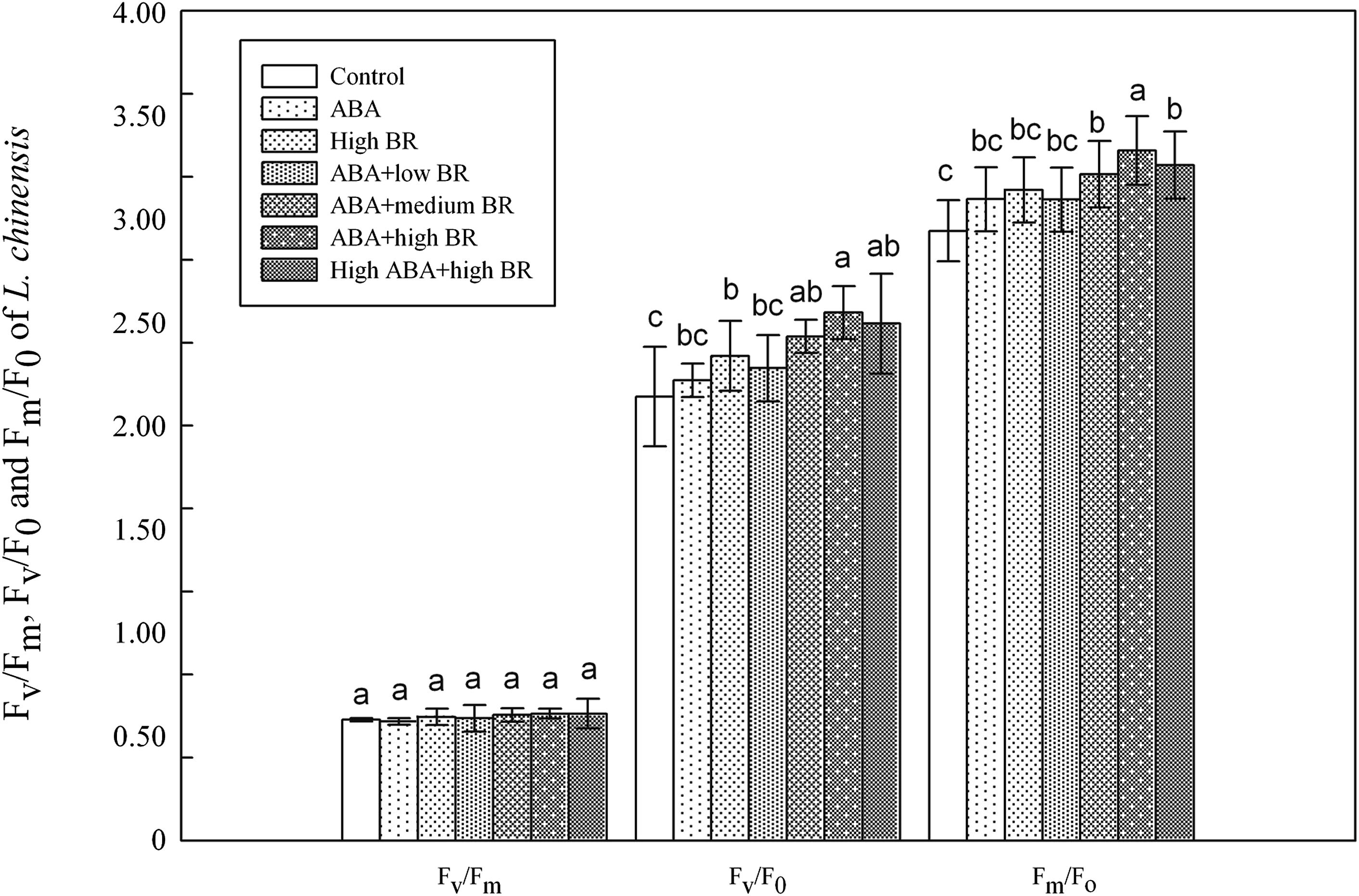

Supplement: Supplementary file 5 — Authors’ original file for figure 5 [file 40529_2011_94_MOESM5_ESM.tiff]
